# Differences in the depression and burnout networks between doctors and nurses: evidence from a network analysis

**DOI:** 10.1186/s12889-024-19193-3

**Published:** 2024-06-22

**Authors:** Zheng Zhang, Hui Chen, Xuting Li, Shurui Chen, Ziyu Wang, Jiaxin Yang, Zengyu Chen, Xiaoping Wang, Yusheng Tian, Jiansong Zhou

**Affiliations:** 1 Department of Psychiatry, National Clinical Research Center for Mental Disorders, and National Center for Mental Disorders, The Second Xiangya Hospital of Central South University, No.139, Renmin Road Central, Changsha, Hunan 410011 China; 2 Clinical Nursing Teaching and Research Section, The Second Xiangya Hospital of Central South University, No.139, Renmin Road Central, Changsha, Hunan 410011 China

**Keywords:** Healthcare professionals, Depression, Occupational burnout, Network analysis

## Abstract

**Background:**

Previous studies have demonstrated a strong association between depression and job burnout among healthcare professionals, but the results have been inconsistent, and there is a lack of in-depth exploration of such a relationship among different healthcare professions. The present study aims to investigate the interrelationships between depression and burnout among Chinese healthcare professionals and whether there are differences in the networks of these symptoms between doctors and nurses.

**Methods:**

The Maslach Burnout Inventory-General Survey and the 2-item Patient Health Questionnaire were employed to assess job burnout and depression among 3,684 healthcare professionals. The translation has been refined to ensure accuracy and academic suitability. Subsequently, network analysis was conducted on 2,244 participants with a higher level of job burnout to identify core symptoms and explore the associations between job burnout and depression.

**Results:**

The present study showed a network association between *lack of interest and pleasure in things* and *being exhausted from work*, *excessive tiredness facing work*, *tendency to collapse at work*, and *lack of passion for work than before* among healthcare professionals, as well as a notable difference in the network association between *lack of interest and pleasure in things* and *lack of passion for work than before* between nurses and doctors.

**Conclusions:**

The depression-burnout network structures differ between doctors and nurses, highlighting the need for targeted intervention measures for both groups.

**Supplementary Information:**

The online version contains supplementary material available at 10.1186/s12889-024-19193-3.

## Introduction

In recent years, the mental health of healthcare workers has become a major concern in China [24]. Several meta-analyses have highlighted the alarming risk of mental health problems faced by healthcare professionals, including depression, anxiety, and insomnia [14, 36]. Depression and job burnout have a significant impact on healthcare professionals worldwide, with reported prevalence rates ranging from 26.2% to 52% for depression and 7.47% to 33.48% for burnout [33, 44, 45]. The World Health Organization (WHO) has classified burnout as an occupational phenomenon in the 11th revision of the International Classification of Diseases (ICD-11) and defines it as a syndrome that has not been successfully managed due to prolonged work stress [17]. These mental health issues can lead to changes in daily life, poor work performance, and reduced quality of patient care, ultimately affecting the healthcare system and society as a whole [48]. It is worth emphasizing that this study was conducted before the COVID-19 pandemic, and it shows higher levels of depression and burnout among healthcare workers during and after the pandemic, with burnout rates reaching more than 50% [6, 43, 46]. Therefore, it is crucial to address the mental health issues of healthcare professionals, especially depression and job burnout, to improve their well-being as well as patient outcomes.

Depression, a mental health problem characterized by low mood, persistent sadness, loss of interest in activities, lack of energy, and sleeping disturbance, is prevalent among healthcare workers [25]. Contributing factors of depression include high workload, long working hours, lack of social support, and exposure to traumatic events [38]. Burnout is another common issue experienced by healthcare workers,it is characterized by emotional, mental, and physical exhaustion due to excessive and prolonged stress [11], and may affect the sense of achievement, sense of control, and work performance of healthcare workers [26]. Taken together, the two conditions have similar and overlapping presentations [40].

Prior studies have demonstrated a strong association between depression and burnout [16]. The job demand-resource (JD-R) model posits that high job demands may lead to negative consequences, such as physical and mental health problems and job burnout, which can be buffered by job resources [9]. In healthcare settings, both doctors and nurses face high levels of job demands, but the nature of their work and the resources accessible to them vary greatly [1]. Doctors may have more autonomy and control over their work, as well as greater access to resources such as support staff and technology. Nurses, on the other hand, may have less control over their work, fewer available resources, and longer work hours [47]. However, another study found no significant difference in burnout between doctors and nurses but highlighted the disparity in resources accessible to the two types of healthcare professionals [32]. Exploring the differences in the relationship between depression and job burnout among different occupational groups using the job demands-resources model is of great significance in providing targeted psychological interventions or preventive measures for these healthcare professionals.

Numerous studies have investigated job burnout and depression at the factor level or variable level, primarily using the perspective of the latent variable model [10, 16]. This perspective helps us understand the internal mechanism of burnout through a simpler and more abstract approach. However, the latent variable model overlooks the specific associations and interactions between symptoms [39]. Borsboom [3] proposed the network theory of mental disorders, an innovative model of psychopathology that re-conceptualizes previous psychological disorders and symptoms. The theory suggests that job burnout, as a psychological disorder affecting people’s physical and mental health, can be viewed as a network of symptoms [27]. Several symptoms in a network system can influence and result in other symptoms in the same network. Previous studies have shown that the network analysis approach has unique advantages in exploring the internal structure of mental health problems [12], which enables us to reveal the relationships between symptoms in a flat manner [4]. For instance, a network analysis of healthcare workers found that depression was significantly associated with symptoms such as high blood pressure, while job burnout, especially the fatigue dimension, was linked to an increased risk of physical illnesses [35]. Thus, network analysis can provide us with more information about interactions at the symptom level compared to the latent variable model [5].

Therefore, we hypothesized that the relationship between depression and job burnout among healthcare professionals differs between nurses and doctors, as shown by the job demands-resources model. This study fills a critical gap by using network analysis to examine these symptom-level interactions. Identifying specific symptoms linking depression and burnout allows for precise interventions tailored to doctors’ and nurses’ unique needs. This research has significant theoretical and practical value, enhancing our understanding of the depression-burnout interplay and providing a foundation for targeted mental health strategies. These improvements can enhance healthcare professionals’ well-being and performance, leading to better patient care and overall healthcare system efficiency, ultimately benefiting society.

## Materials and methods

### Participants

The survey was conducted on frontline healthcare workers using a convenience sampling method (snowball sampling) from January 10 to February 5, 2019. The questionnaires were distributed through Wenjuanxing, an online platform, and each IP address was allowed to submit the answers only once. The survey was shared on WeChat by 40 healthcare workers who were initially involved in this study. A total of 3,706 responses were received from 23 provinces, and after removal of invalid responses, a total of 3,684 responses were included in the final analysis. Quality control measures, such as screening for submitted responses with too short completion time (less than 240 s) and the use of an attention-detection question (selecting the second option for this question), were implemented. The study was approved by the ethics committee of the Ethics Committee of the Xiangya Second Hospital of Central South University, and all participants signed informed consent online before participating.

### Measurement

#### Maslach burnout inventory-general survey

The Chinese version of Maslach Burnout Inventory-General Survey (CMBI-GS) was used to assess the job burnout of participants. The CMBI-GS consists of five items that assess emotional exhaustion (e.g., How often you being exhausted from work), four items that measure depersonalization (e.g., How often you lack of passion for work than before), and six items that measure personal achievement (e.g., How often you being confident in completing various tasks) [19]. All items and explanations for CMBI-GS are presented in Supplementary Table S1. Each item was rated on a 7-point Likert scale from 0 to 6. The score for each of the three factors was calculated as the average score of all items included in the corresponding dimension (i.e., the total score divided by number of items). All the participants were divided into a high-burnout group (with a CMBI-GS score of 8.5–18) and a low-burnout group (with a CMBI-GS score of 0–8.5). With higher scores indicating greater burnout levels [8]. The Cronbach’s alpha of this scale was 0.870 in the present study.

#### The 2-item patient health questionnaire

The 2-item Patient Health Questionnaire (PHQ-2) consists of the first two items of the PHQ-9 and evaluates the frequency of depressed mood and lack of interest/pleasure in activities over the past two weeks (e.g., How often have you felt lack of interest and pleasure in things in the past two weeks, and how often have you felt down, depressed, or hopeless in the past two weeks [23]. All items and explanations for PHQ-2 are presented in Supplementary Table S1. Each item is rated on a 4-point Likert scale from 0 to 3, and the total score ranges from 0 to 6. The results were determined as negative (0–4) and positive (5–6) based on the corresponding criteria. For this scale, higher scores indicate greater levels of depression. The Cronbach’s alpha of this scale was 0.824 in the present study.

### Statistical analyses

The data analysis in the present study comprises four main steps, with each step built upon the previous one. Firstly, data were processed using SPSS22.0, which involved missing value deletion, variable assignment, and reclassification. Secondly, descriptive statistics were compiled and t-tests performed using JASP 0.16.2 to identify any significant differences between variables [20]. Thirdly, partial correlation analysis was conducted with the controlling for gender, marital status, education background, smoking, alcohol consumption, occupation, and length of service to examine the relationship between variables in greater depth. Finally, network analysis was performed in three steps: network structure estimation, network description (including node centrality and network comparisons), and network stability analysis [5]. For symptom-level network analysis, individual symptoms were represented by nodes, and the relationship between symptoms were represented by edges. Network and centrality graphs were generated to visualize the symptom network.

To investigate the variations in depression and job burnout networks among healthcare workers with distinct occupational types, we divide the healthcare workers with a high level of burnout into two groups based on their professions. We then constructed network analysis and centrality diagrams to examine the stability of the network structure and centrality for each group. The centrality of network reflects the importance of nodes based on their location in the network. Some commonly used indicators for centrality include 1) degree centrality, which indicates the connections of a node regardless of the importance, 2) betweenness centrality, which identifies nodes that are critical for information flow but is computationally expensive, 3) closeness centrality, which measures the distance of a node to other nodes but can be affected by isolated nodes or clusters, and 4) expected influence, which measures the potential impact of a node on other nodes but is difficult to calculate and dependent on assumptions about node behaviour. This approach was inspired by previous studies that utilized network analysis to examine the relationships between symptoms and mental disorders [41].

Overall, the present study used a systematic and comprehensive approach for data analysis, starting with data processing and moving on to more sophisticated techniques such as network analysis. These procedures enabled us to improve our understanding of the complex relationships between variables and identify any significant differences between groups.

## Results

### Demographic characteristics

A total of 3,684 healthcare workers were included in this study, including 934 doctors and 2,750 nurses, aged from 18 to 72 years (mean age = 31.63 ± 7.69 years). Additional demographic and occupational information were presented in Supplementary Table S2. A total of 2,247 respondents reported a high level of job burnout (60.99%), including 524 doctors and 1,723 nurses, with an average age of 30.91 ± 7.17 years. Additional demographic information is presented in Table 1. Then the differences in PHQ-2 and CMBI-GS scores between doctors and nurses in the high-burnout group were analyzed by independent samples t-test. The results showed that the score of PHQ-2 was 2.58 ± 1.68 for doctors and 2.67 ± 1.61 for nurses, and no significant difference was found between the two types of professionals (*t* = 1.08, *p* = 0.28). The score of CMBI-GS was 12.04 ± 2.34 for doctors and 12.11 ± 2.37 for nurses, and no significant difference was found between the two types of professionals (*t* = 0.60, *p* = 0.55).

**Table 1:** Demographic characteristics of healthcare workers with a high level of burnout

Variable	Doctors (*n* = 524, n [%])	Nurses (*n* = 1,723, n [%])	χ^2^
Gender			
Male	233 (44.47)	95 (5.51)	489.024^***^
Female	291 (55.53)	1628 (94.49)	
Marital status			
Unmarried	158 (30.15)	664 (38.54)	12.176^***^
Married	366 (69.85)	1059 (61.46)	
Education background			
Junior college and below	39 (7.44)	438 (25.42)	295.732^***^
Bachelor’s degree	325 (62.02)	1195 (69.36)	
Master’s degree or above	160 (30.54)	90 (5.22)	
Smoking status			
Non-smoker	411 (78.44)	1617 (93.85)	108.511^***^
Smoker	113 (21.57)	106 (6.15)	
Alcohol consumption			
Non-drinker	245 (46.76)	1150 (66.74)	68.196^***^
Drinker	279 (53.24)	573 (33.26)	
Length of service (year)			
< 5	181 (34.54)	703 (40.80)	65.205^***^
6–10	113 (21.57)	569 (33.02)	
11–20	147 (28.05)	311 (18.05)	
> 20	83 (15.84)	140 (8.13)	
Hospital Type			67.856^***^
Primary Hospital	35 (6.68)	29 (1.68)	
Secondary Hospital	123 (23.47)	502 (29.14)	
Tertiary Hospital	366 (69.85)	1192 (69.18)	
Professional Title			203.137^***^
Senior	118 (22.52)	87 (5.05)	
Intermediate	169 (32.25)	421 (24.43)	
Junior	237 (45.23)	1215 (70.52)	
Shift Work			20.216^***^
Yes	122 (23.28)	327 (18.98)	
No	402 (76.72)	1396 (81.02)	
Years of Service			79.198^***^
< 5 years	181 (34.54)	703 (40.8)	
6–10 years	113 (21.56)	569 (33.02)	
11–20 years	147 (28.05)	311 (18.05)	
20 years	83 (15.84)	140 (8.13)	
Working Hours			324.163^***^
50 h/week	202 (38.55)	172 (9.98)	
40–50 h/week	249 (47.52)	1081 (62.74)	
< 40 h/week	73 (13.93)	470 (27.28)	

^***^p < 0.01

### Correlation analysis

**Fig. 1:**
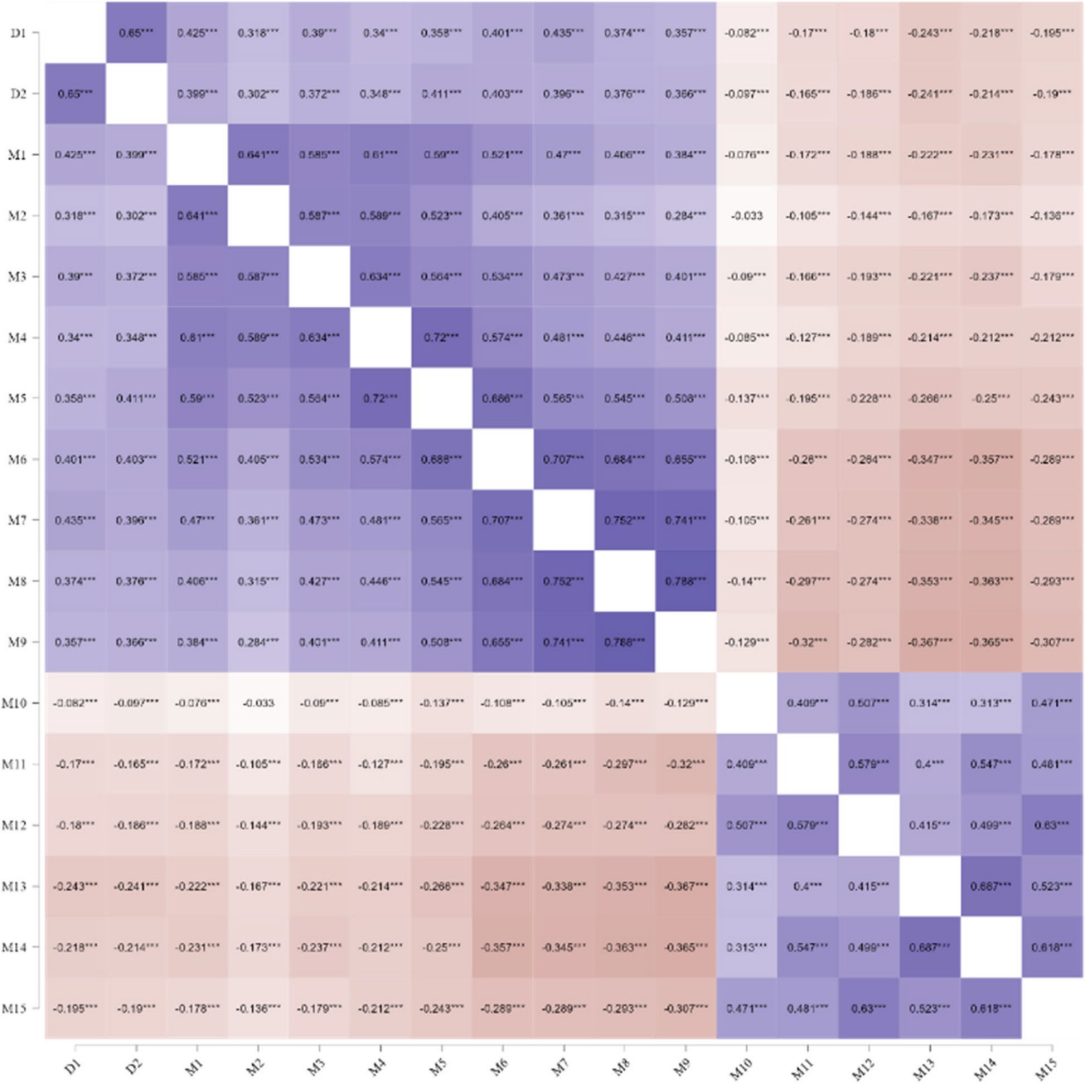
Heat map of the correlation between symptoms of depression and burnout. Notes: In the network, positive correlations are represented by purple lines, while negative correlations are shown in red. The intensity of the color reflects the strength of the correlation, with darker shades indicating higher levels of correlation

In the correlation analysis, gender, marital status, education background, smoking status, alcohol consumption, occupation, and length of service were used as control variables, and the correlation coefficients of all items were included in the statistical analysis to generate partial correlation heat maps (Fig. 1). A close relationship was found between job burnout and depression, with dimensions of emotional exhaustion and depersonalization being positively correlated with depression while the dimension of sense of achievement was negatively correlated with depression.

### Network analysis: relationship between depression and job burnout in healthcare workers

**Fig. 2:**
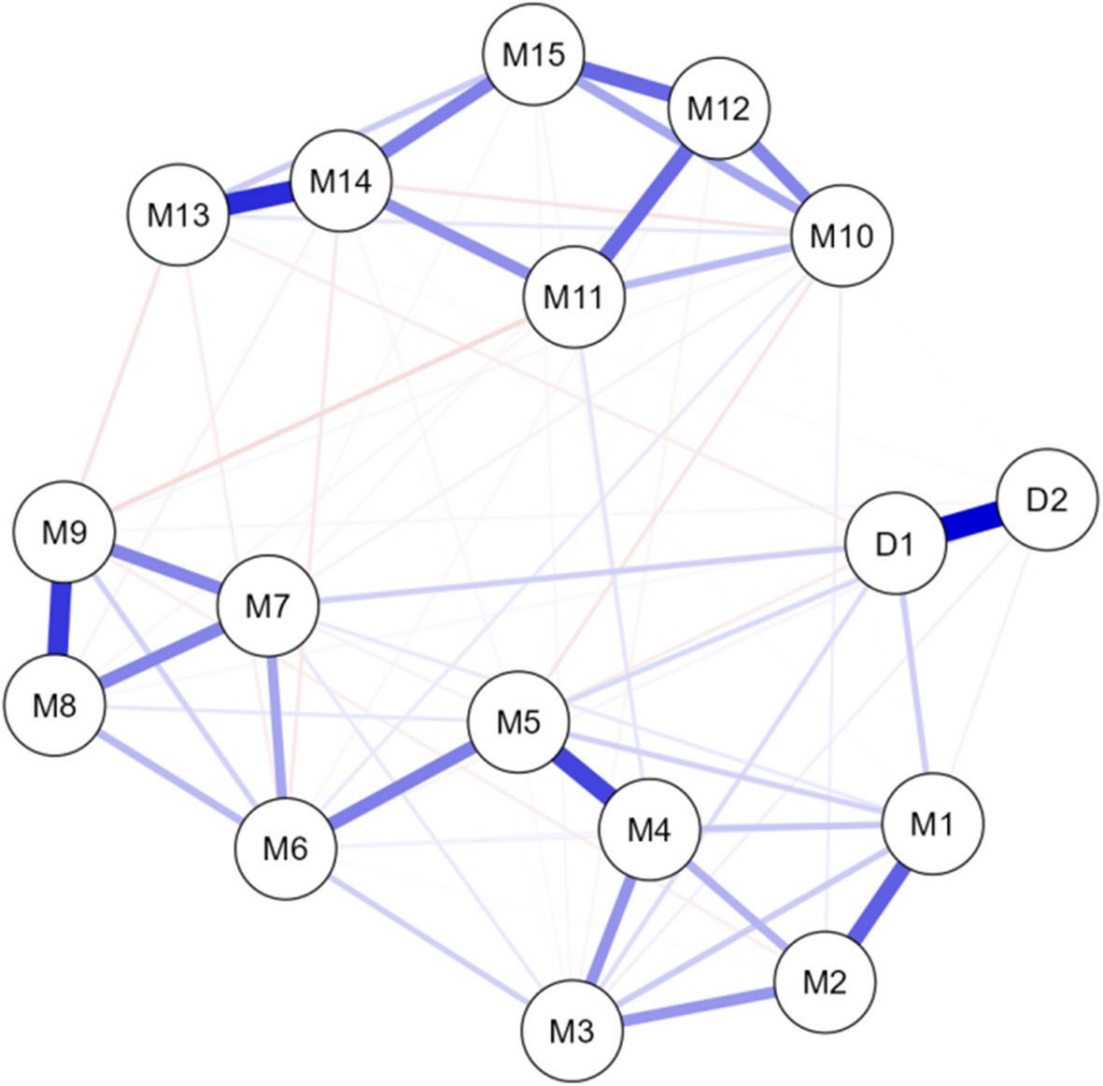
The depression and burnout network of healthcare workers. Note: In this network, each node represents an item (D for depression and M for burnout). Positive correlations are indicated using blue lines, and negative correlations are marked in red lines. The thickness of the lines reflects the strength of the correlation

The number of non-zero edges in the network of depression and job burnout among all the healthcare professionals was 76/136, with a sparsity of 0.441. The two symptoms of depressive disorder had a high correlation (0.599). A certain correlation was found between depression (*lack of interest and pleasure in things*) and job burnout network (*being exhausted from work, excessive tiredness facing work, tendency to collapse from work, lack of passion for work than before*). The depression and burnout network of healthcare workers is presented in Fig. 2, and the robustness of this network is shown in Supplementary Figure S1.

### Comparison of the depression and burnout network between doctors and nurses

To further examine the differences in the depression and job burnout network between doctors and nurses, we divided the high-burnout group into two subgroups based on their occupation; network analysis diagrams and centrality diagrams were then created, and the stability of network structure and centrality were tested.

### Network structure estimation

**Fig. 3:**
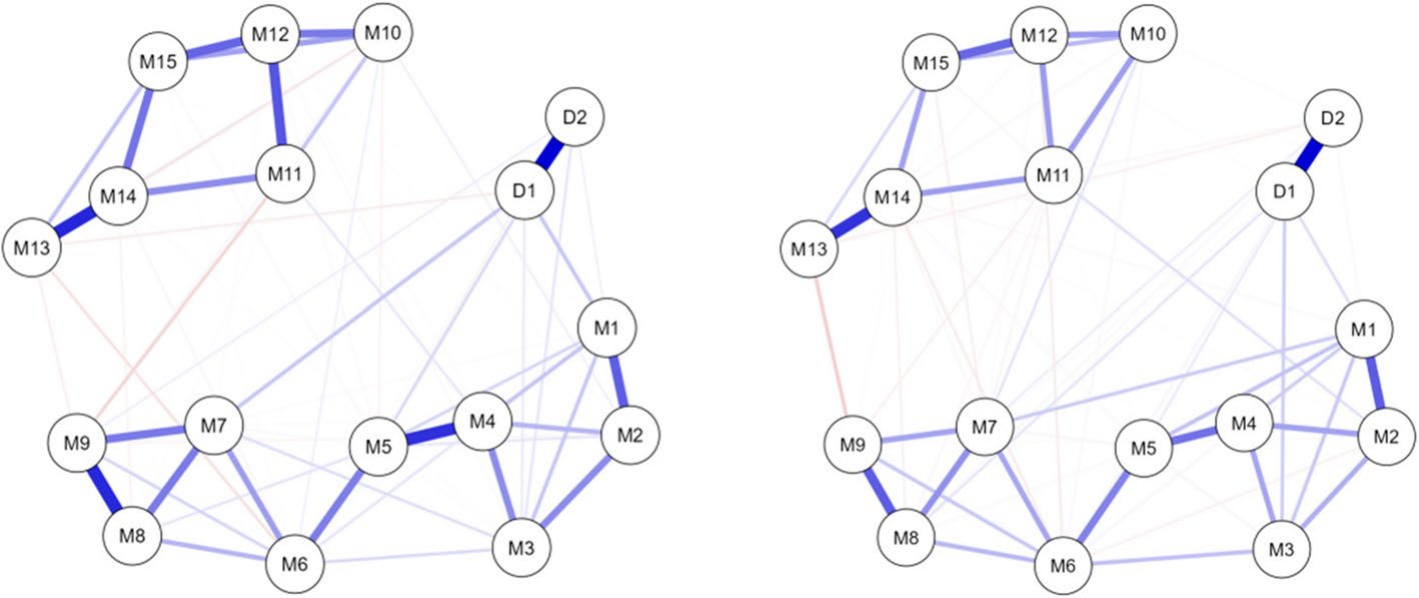
Comparison of network connections between nurses (left) and doctors (right). Note: Nodes in the network on the left represent nurses, and nodes in the network on the right represent doctors

The Number of non-zero edges of the depression and job burnout network for nurses was 73/136, with a sparsity of 0.463; the number of non-zero edges of depression and job burnout network for doctors was 70/136, with a sparsity of 0.485. The depression-burnout networks of doctors and nurses are presented in Fig. 3, and the robustness of the two networks is displayed in Supplementary Figure S2. Overall, the depression and job burnout network for doctors appeared to be looser and less connected, but the difference between the two types of professionals was not significant.

A strong connection between *lack of interest and pleasure in things* and *lack of passion for work than before* (0.12) was found in the nurse group, while such a connection appeared weaker among doctors (0.01), suggesting that there might be a stronger association between interest in life and passion for work among nurses. The correlation between *Excessive stress at work* &*Tendency to collapse from work.* was stronger among nurses (0.462 > 0.356), indicating that nurses might be more likely to experience nervous breakdown under excessive pressure.

### Node centrality

**Fig. 4:**
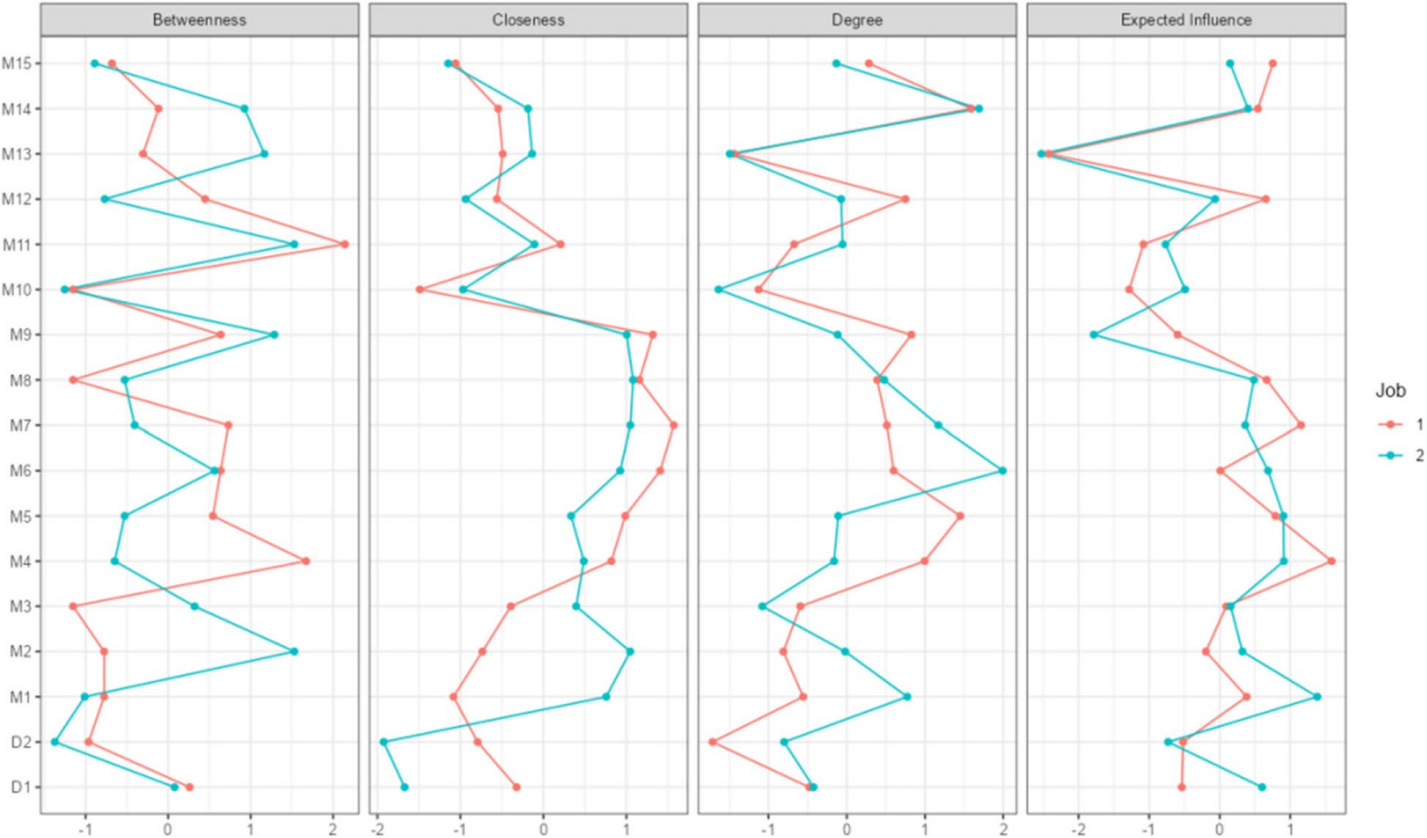
The centrality diagrams of the depression and burnout networks. Note. Centrality refers to the relative importance of a node within the network. In this case, nodes with a centrality value of 1 represent nurses, and nodes with a value of 2 represent doctors

The present study focused on betweenness centrality when analyse the differences. The centrality of nodes in the depression-burnout network of doctors and nurses is presented in Fig. 4, and the robustness of the centrality indicators is shown in Supplementary Figure S3.

With regard to nurses, the two nodes with high centrality indicators were *being exhausted from work* and excessive *stress at work*; for doctors, the highest centrality was found in the node *making a great contribution to hospital* (1.531). In addition, the centrality of *feeling drained after work* (1.531) was also high, which indicated indifference to contributions and exhaustion after work.

## Discussion

The present study indicated that there is no significant difference in the overall scores of job burnout and depression between doctors and nurses. Correlation analysis showed that emotional exhaustion and depersonalization were positively correlated with depression, while the sense of achievement was negatively correlated with depression. Network analysis revealed differences in the burnout-depression network structures between doctors and nurses, as well as differences in the core symptoms. These findings underscore the importance of targeted intervention measures for different healthcare professions, which can enhance the mental health and job satisfaction of healthcare workers, ultimately improving patient care quality and the overall effectiveness of the healthcare system.

Our study found that the detection rate of high level of job burnout among Chinese healthcare professionals was 60.99%. Compared to healthcare workers in other countries, Chinese healthcare workers experience a moderate level of burnout and depression [22]. Mosolova et al. [31] found that the detection rate of depression and burnout among Russian healthcare workers was 45.5% and 37.7–74.2%, respectively, which is much higher than that in China. Similarly, Guttormson et al. [15] reported that 44.6% of nurses in the United States had moderate to severe depressive symptoms. In contrast, Dobson et al. [10] found that the prevalence of depression and burnout among healthcare workers in New Zealand was about 21% and 29.5%, respectively. Thus, occupational burnout is an important issue affecting the mental health of healthcare workers worldwide, which necessitates the development of interventions for this problem.

Depression and burnout are interrelated, as supported by existing studies [7, 40]. Healthcare professionals are particularly susceptible to depression and burnout due to a variety of risk factors such as high workload, their emotional demanding role, and low job control. Investigation of the characteristics of depression and burnout among different types of healthcare providers can facilitate the development of targeted interventions to improve their mental health and well-being. Prior studies have also explored the differences in depression and burnout between doctors and nurses [10]. Consistent with the findings of See et al. [37], burnout was found to be significant in both doctors and nurses, with no discernible differences in its level. However, some studies still suggest that the level of burnout may be slightly higher among nurses than among doctors [29]. Therefore, it becomes crucial to further clarify the absolute differences and structural differences of depression and burnout between doctors and nurses.

Through the analysis of network edges, we found differences in the depression-burnout networks between doctors and nurses, which are reflected in the edges and centrality of nodes. Specifically, nurses showed a stronger connection between their interest in life and passion for work as well as a greater tendency to feel overwhelmed under great pressure, and the heavy workload and pressure may lead to a higher risk of burnout. Regarding centrality in the network analysis, we found that the perception of contribution/usefulness (M11) was the most representative node in the burnout and depression network for both doctors and nurses. According to Hatch et al. [18], the perception of contribution and usefulness is a crucial factor in coping with burnout and depression among healthcare workers, especially doctors and nurses. Thus, healthcare facilities need to provide effective staff training that involves the meaning and importance of their work, in order to reduce burnout and depression and promote well-being of their staff members.

Our t-test results showed no significant differences in depression or burnout levels between doctors and nurses in the high-burnout group. From the perspective of the traditional latent variable model, this suggests that the level of depression and burnout is consistent, which is difficult to explain at the moment. However, our network analysis revealed differences in network association between depression and burnout between doctors and nurses, demonstrating the value of network analysis in providing a higher level of granularity to reveal more information about the interrelationship between these two conditions. This is consistent with previous research [39].

According to the JD-R model, job demands and resources have a significant impact on the well-being of employees, as highlighted by Zhou et al. [48]. To reduce depression and burnout among doctors and nurses, healthcare facilities can focus on reducing workload, role conflict, and emotional demands, as well as increasing accessible resources such as social support and opportunities for professional development. Team-based care, mindfulness training, and encouraging feedback are also practical measures to lower the risk of depression and burnout among healthcare workers; these measures may be used alone or in combination, and they have demonstrated great effectiveness, as reported by Aryankhesal et al. [2] Furthermore, promoting work-life balance through flexible work schedule and support for self-care activities may also help to reduce depression and burnout.

Targeted interventions are recommended to improve the mental health of Chinese doctors and nurses due to their unique work environment. Generally, nurses spend far more time with patients compared to doctors, which may lead to emotional exhaustion and burnout. Thus, interventions for nurses need to focus on improving their emotional intelligence, communication skills, and empathy towards patients. For example, empathy training programs and mindfulness-based interventions. Research suggests that empathy training can enhance empathy skills among nurses, potentially reducing emotional distress and burnout [30]. And mindfulness-based interventions can improve self-compassion among healthcare professionals, including nurses, leading to reduced stress and burnout [42].Nurses should also be trained in how to cope with the emotional demands of their job, such as breaking bad news and providing emotional support to patients and their families [21]. In contrast, doctors are believed to have higher professional competence, which leads to higher expectations and pressure from colleagues and patients. Therefore, interventions for doctors need to focus on the development of self-compassion and stress management skills. For example, mindfulness and self-compassion programs and physician wellness programs. Research protocols suggest that mindfulness and self-compassion programs can effectively reduce work stress and burnout among family and community medicine physicians. Comprehensive wellness programs addressing empathy, emotional intelligence, and work-life balance may prove beneficial for physicians in managing stress and preventing burnout [34]. Doctors should be trained in how to balance their professional responsibilities with self-care and stress reduction, such as mindfulness or relaxation exercises [28, 34]. By providing targeted interventions addressing the unique needs of doctors and nurses, healthcare facilities can effectively reduce depression and burnout of their staff [13]. These interventions target key factors contributing to depression and fatigue in both nurse and doctor populations, offering valuable insights into effective strategies for promoting mental health and well-being in healthcare settings.

### Limitations and future direction

It is important to note some limitations of the present study. Firstly, this is a cross-sectional study, which precluded us from explore the causal relationship. Therefore, longitudinal studies are warranted to explore the causal relationship between depression, burnout, and other factors. Secondly, this study only focused on the burnout and depression network of healthcare workers with a high level of job burnout, with a limited number of elements included. Thus, future studies may focus on networks at the factor or variable level to obtain more information on the protective and risk factors of burnout among healthcare workers. Hopefully, this may improve our understanding of the complex relationships between various factors and the development of burnout in healthcare workers.

## Conclusions

At the latent variable level, no differences were observed between doctors and nurses in terms of depression and occupational burnout. However, network analysis revealed differences in the depression-burnout networks of doctors and nurses, including variations in network structures and core symptoms. Therefore, it is imperative to provide targeted intervention measures for both groups.

### Supplementary Information

Supplementary Material 1. See Table S1-S2 and Figures S1-S3 in the Supplementary Material for comprehensive image analysis.

## Data Availability

The data that support the findings of this study are available from the corresponding author, upon reasonable request.
