# Supplementary material for: Differences in the depression and burnout networks between doctors and nurses: evidence from a network analysis
Source: BMC Public Health. 2024 Jun 22;24:1668. doi: 10.1186/s12889-024-19193-3 (PMC11193897; doi:10.1186/s12889-024-19193-3)
Supplement: Supplementary file 1 — Supplementary Material 1. See Table S1-S2 and Figures S1-S3 in the Supplementary Material for comprehensive image analysis. [file 12889_2024_19193_MOESM1_ESM.docx]

**Supplementary Materials**

**Supplementary Table S1.** The meanings of the items in the depression and burnout scales

| M1 | Being exhausted from work. |
| --- | --- |
| M2 | Feeling drained after work. |
| M3 | Excessive tiredness facing work. |
| M4 | Excessive stress at work. |
| M5 | Tendency to collapse from work. |
| M6 | Loss of interested in work. |
| M7 | Lack of passion for work than before. |
| M8 | Doubtfulness about the meaning of work. |
| M9 | Lack of concern about one’s contribution. |
| M10 | Effective problem solving at work. |
| M11 | Making a great contribution to hospital. |
| M12 | Being good at my work. |
| M13 | Feeling happy upon completion of tasks. |
| M14 | Having done a wonderful job. |
| M15 | Being confident in completing various tasks. |
| D1 | Lack of interest and pleasure in things. |
| D2 | Feeling down, depressed, or hopeless. |

Note: M1-M15 are associated with the items in the Occupational Burnout Scale, and D1 and D2 are related to the two items in the PHQ-2.

**Table S2.** Demographic and occupational characteristics of healthcare workers

| Variable | Doctors  (n=934, n [%]) | Nurses  (n=2,750, n [%]) | χ^2^/t |
| --- | --- | --- | --- |
| Gender |  |  | 733.75*** |
| Male | 397 (42.51) | 159 (5.78) |  |
| Female | 537 (57.49) | 2591 (94.22) |  |
| Age Group |  |  | 153.34*** |
| <30 years | 324 (34.69) | 1456 (52.95) |  |
| 30-39 years | 350 (37.47) | 956 (34.76) |  |
| ≥40 years | 260 (27.84) | 338 (12.29) |  |
| Marital status |  |  | 9.54*** |
| Unmarried | 281 (30.09) | 980 (35.64) |  |
| Married | 653 (69.91) | 1770 (64.36) |  |
| Education background |  |  | 380.33*** |
| Junior degree or below | 93 (9.96) | 693 (25.2) |  |
| Bachelor’s degree | 573 (61.35) | 1888 (68.65) |  |
| Master's degree or above | 268 (28.69) | 169 (6.15) |  |
| Work schedule |  |  | 20.22*** |
| Non-shift | 302 (32.33) | 682 (24.80) |  |
| Shift | 632 (67.67) | 2068 (75.20) |  |
| Smoking status |  |  | 178.94*** |
| Non-smoker | 739 (79.12) | 2588 (94.11) |  |
| Smoker | 484 (51.82) | 162 (5.89) |  |
| Alcohol consumption |  |  | 96.36*** |
| Non-drinker | 450 (48.18) | 1822 (66.25) |  |
| Drinker | 484 (51.82) | 928 (33.75) |  |
| Level of hospital |  |  | 67.86*** |
| Tertiary hospital | 625 (66.92) | 1916 (69.67) |  |
| Secondary hospital | 226 (24.20) | 761 (27.67) |  |
| Primary/community hospital | 83 (8.89) | 73 (2.65) |  |
| Professional title |  |  | 203.14*** |
| Senior | 211 (22.59) | 183 (6.65) |  |
| Mid-Level | 273 (29.23) | 748 (27.20) |  |
| Junior | 450 (48.18) | 1819 (66.15) |  |
| Length of service (year) |  |  | 79.20*** |
| <5 | 341 (36.51) | 1041 (37.85) |  |
| 6-10 | 183 (19.59) | 871 (31.67) |  |
| 11-20 | 233 (24.95) | 533 (19.38) |  |
| >20 | 177 (18.95) | 305 (11.09) |  |
| Working Hours |  |  | 324.16*** |
| >50h/week | 301 (32.23) | 231 (8.4) |  |
| 40-50h/week | 452 (48.39) | 1688 (61.38) |  |
| <40h/week | 181 (19.38) | 831 (30.22) |  |
| Burnout level |  |  | 12.58*** |
| Low | 410 (43.9) | 1027 (37.35) |  |
| High | 524 (56.1) | 1723 (62.65) |  |
| The total score of PHQ-2 (M±SD) | 1.92±1.65 | 2.10±1.63 | 2.92*** |
| The total score of CMBI-GS (M±SD) | 9.37±3.77 | 9.87±3.66 | 3.55*** |

Note: *** *p* <0.01; PHQ-2: The 2-item Patient Health Questionnaire; CMBI-GS: The Maslach Burnout Inventory-General Survey.


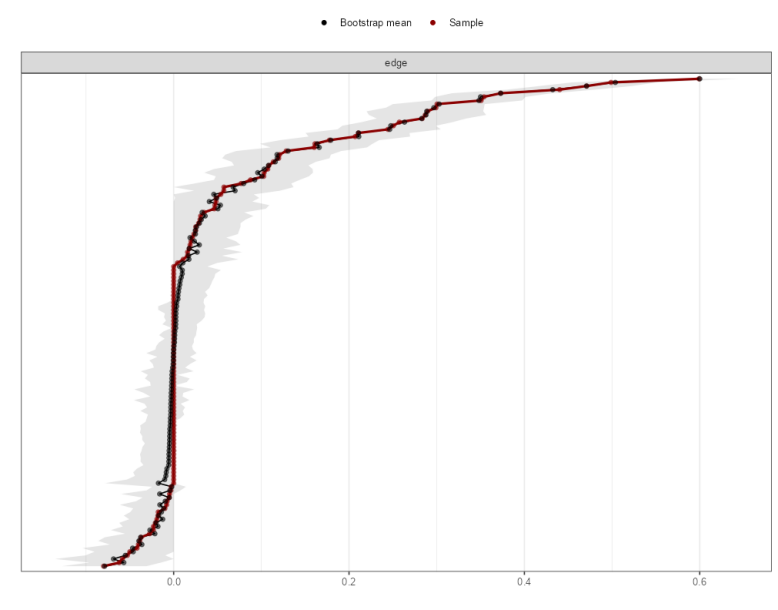


**Supplementary Figure S1.** Robustness of network structure

The gray band of Supplementary Figure 1 is the range of changes in network connectivity, with narrower gray range indicating a more robust depression-versus-burnout network structure.


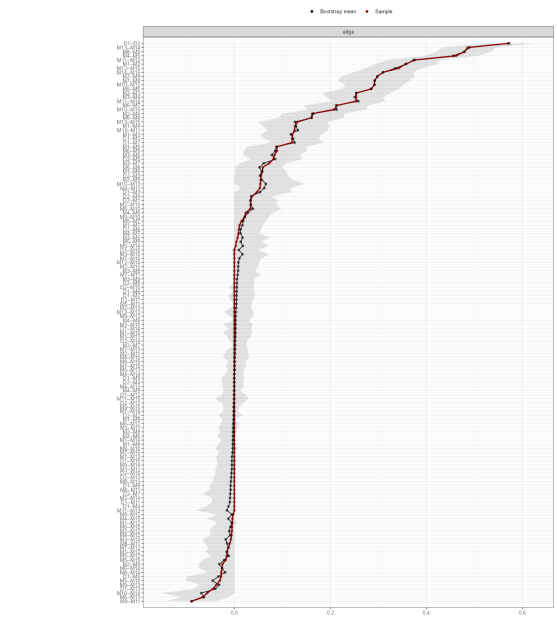

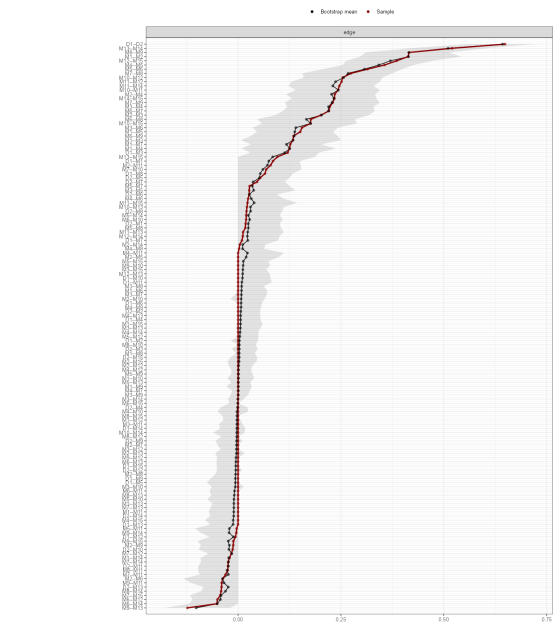


**Supplementary Figure S2.** Robustness of network structure for nurses (left) and doctors (right)


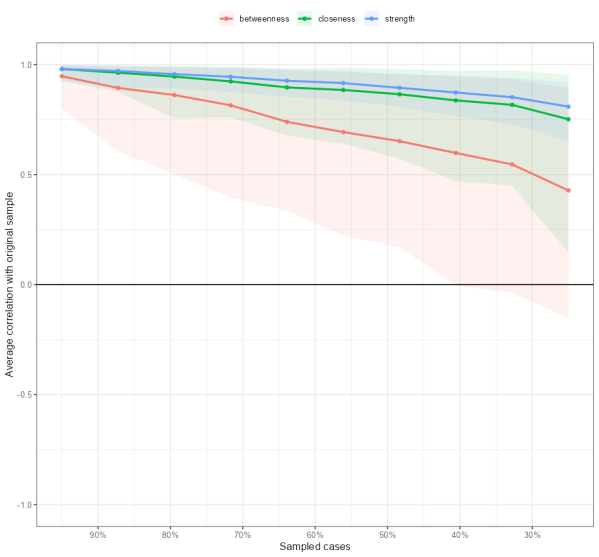

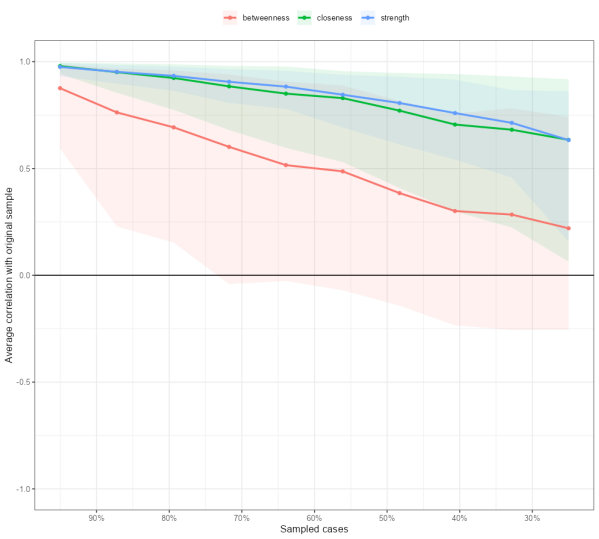


**Supplementary Figure S3.** Robustness of network centrality for nurses (left) and doctors (right).

The centrality was deemed stable when it remained little changed with the decrease of sample size.
